# Interleukin-17A and Neutrophils in a Murine Model of Bird-Related Hypersensitivity Pneumonitis

**DOI:** 10.1371/journal.pone.0137978

**Published:** 2015-09-14

**Authors:** Masahiro Ishizuka, Yasunari Miyazaki, Masahiro Masuo, Kozo Suhara, Tomoya Tateishi, Makito Yasui, Naohiko Inase

**Affiliations:** Department of Respiratory Medicine, Tokyo Medical and Dental University, Tokyo, Japan; Louisiana State University, UNITED STATES

## Abstract

Hypersensitivity pneumonitis (HP) is an immune mediated lung disease induced by the repeated inhalation of a wide variety of antigens. Bird-related hypersensitivity pneumonitis (BRHP) is one of the most common forms of HP in human and results from the inhalation of avian antigens. The findings of a recent clinical analysis suggest that in addition to Th1 factors, the levels of interleukin(IL)-17 and IL-17-associated transcripts are increased in the setting of HP, and that both IL-17A and neutrophils are crucial for the development of pulmonary inflammation in murine models of HP. Our objectives were to investigate the roles of IL-17A and neutrophils in granuloma-forming inflammation in an acute HP model. We developed a mouse model of acute BRHP using pigeon dropping extract. We evaluated the process of granuloma formation and the roles of both IL-17A and neutrophils in a model. We found that the neutralization of IL-17A by the antibody attenuated granuloma formation and the recruitment of neutrophils, and also decreased the expression level of chemokine(C-X-C motif) ligand 5 (CXCL5) in the acute HP model. We confirmed that most of the neutrophils in the acute HP model exhibited immunoreactivity to the anti-IL-17 antibody. We have identified the central roles of both IL-17A and neutrophils in the pathogenesis of granuloma formation in acute HP. We have also assumed that neutrophils are an important source of IL-17A in an acute HP model, and that the IL-17A-CXCL5 pathway may be responsible for the recruitment of neutrophils.

## Introduction

Hypersensitivity pneumonitis (HP) is an immune mediated lung disease induced by the inhalation of a wide variety of antigens [1]. Bird-related hypersensitivity pneumonitis (BRHP) is one of the most common forms of HP and results from the inhalation of avian antigens [2]. The presence of specific-IgG antibodies in most cases of HP suggests that a type III hypersensitivity mechanism may be responsible for the disease’s underlying pathology. Although a type III hypersensitivity mechanism has been pathophysiologically proposed, it is currently believed that a type IV hypersensitivity mechanism mediated by T cells may also be involved [3].

There appear to be two types of HP based on the disease’s clinical features, as follows: acute and chronic [4]. The symptoms of acute HP occur 4 to 6 h following an exposure to an etiologic antigen and consist of the abrupt onset of a flu-like syndrome characterized clinically by fever, chills, malaise, and myalgias [5]. The respiratory symptoms include severe dyspnea, chest tightness, and a nonproductive cough. The clinical features of acute BRHP include the reproduction of symptoms following exposure to an avian antigen, the presence of specific IgG or IgA antibodies to PDE in the sera and BAL fluid, the proliferation of either BAL or peripheral lymphocytes in response to pigeon sera, the finding of lymphocytosis in the BAL fluid, and the presence of both alveolitis and granulomatous lesions in the lungs [6,7].

A well-described murine model of HP induced by the repeated intratracheal or intranasal administration of *Saccharopolyspora rectivirgula* (SR) antigen which is the causative agent in farmer’s lung develop mononuclear infiltrates in a peribronchovascular distribution in C57BL/6 mice same as the human disease [8–12]. The bronchoalveolar lavage (BAL) fluids from the SR antigen-challenged mice are characterized by copious quantities of neutrophils. It is known that neutrophils and T cells produce interferon(IFN)-γ, a cytokine important in the development of HP [13]. Th1-prone C57BL/6 mice are more susceptible to HP than Th2-prone DBA/2 mice following exposure to SR [14]. Several groups have reported that Th1 mediators such as IFN-γ, interleukin(IL)-12, and chemokine(C-C motif) ligand 3 (CCL3) are present in the lungs of mice challenged with SR antigen [15,16], and that IFN-γ deficient mice are protected from developing HP [17]. Mice overexpressing GATA binding protein 3 (GATA-3), a transcription factor required for Th2 differentiation, are protected from HP because of the suppression of the Th1 response [18].

The findings of a recent clinical analysis suggest that in addition to the Th1 factors, the levels of IL-17 and IL-17-associated transcripts are increased in the setting of clinical HP [19]. The gene deletion of either IL-17 or the IL-17 receptor, as well as the *in vivo* neutralization of IL-17 in an experimental model of HP driven by repeated SR antigen challenges, results in protection from HP, indicating that IL-17 are necessary for the development of mononuclear infiltrates in this model [12,20].

In the present study, we developed an acute HP model, which involved the intratracheal spraying of PDE into C57BL/6 mice three times a week for 10 days, and investigated the role of IL-17A in granuloma-forming inflammation in the setting of acute HP, using an anti-mouse IL-17A antibody.

## Materials and Methods

### Mice

Specific-pathogen-free female C57BL/6 mice were purchased from Sankyo Medical Animal Supply (Sankyo Lab. Co., Tokyo, Japan). The mice were bred in the animal facility of the Tokyo Medical and Dental University under specific-pathogen-free conditions and included in the study after reaching 8 weeks of age. All animal experiments were approved by the Institutional Animal Care and Use Committee of Tokyo Medical and Dental University (Permit Number: 0150028C).

### The preparation of pigeon dropping extract (PDE)

The pigeon dropping extract (PDE) was obtained as previously described [21]. Briefly, fresh pigeon droppings were stirred with a 20-fold volume of phosphate buffered saline solution (pH 7.4) for 24 hours and then dialyzed with distilled water. The extract was then sterilized via filtration (Millex-GV; Millioire, Bedford, MA, USA) and lyophilized.

### The development of acute HP

The mice in both the control group and the study group were immunized and boosted via an intraperitoneal injection of 8 μg of PDE absorbed onto an alhydrogel (alum; Pierce, Rockford, IL, USA) as previously described [22]. They were then placed under light anesthesia with 100 mg/kg ketamine and 10 mg/kg xylazine, and 5 μg of PDE in 25 μl of 0.9% saline was sprayed into each mouse’s trachea using an intratracheal MycroSprayer® aerosolizer (Penn-century, Wyndmoor, PA, USA). Spraying was conducted on days 0, 2, 4, and 7. The control mice were sprayed with 0.9% saline in the same manner in which the PDE was administered to the mice in the study groups.

**Fig 1:**
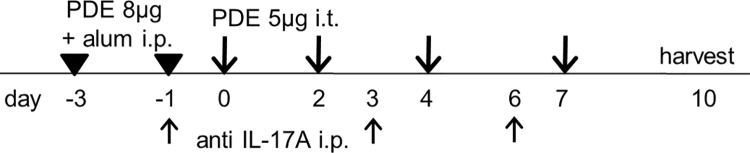
A schematic representation of a timeline for the administration of avian antigens and antibodies. The mice were immunized and boosted via the intraperitoneal injection of 8 μg of pigeon dropping extract (PDE) absorbed onto an alhydrogel (alum), both three days before (day −3) and one day before (day −1) beginning the administration of PDE. The mice were each challenged with 5 μg of PDE on days 0, 2, 4, and 7. In some of the experiments, 100 μg of anti-interleukin(IL)-17A antibody was injected intraperitoneally on days −1, 3, and 6. Both the bronchoalveolar lavage (BAL) and lung specimens were harvested on days 0, 2, 4, 7, and 10.

In order to neutralize IL-17A, 100 μg of rat anti-mouse IL-17A monoclonal antibody (clone 50104; R&D Systems, Minneapolis, MN, USA) was injected intraperitoneally into each mouse on days −1, 3, and 6. A total of either three anti-IL-17A injections or three control IgG injections were administered per mouse. The timeline for PDE and anti-IL-17A administration is shown in Fig 1.

### Bronchoalveolar lavage (BAL) and flow cytometric analysis

After the mice were euthanized by ketamine-xylazine overdose, a plastic cannula was inserted into the trachea. Bronchoalveolar lavage samples were obtained by thrice washing each pair of lungs with 1.0-ml aliquots of 0.9% saline. Following centrifugation, the bronchoalveolar lavage cell pellets were washed and resuspended in phosphate buffered saline (PBS), and the total cell counts were determined using a portion of the suspension. The cytospin preparations were fixed and stained using Diff-Quick (Dade, Behring, Deerfield, IL, USA), and the differential cell counts were analyzed. In the remaining suspension, erythrocytes were lysed using red blood cell lysis buffer. The remaining cells were washed and incubated with anti-CD16/32 in order to block the Fc receptors (Tonbo Bioscience, San Diego, CA, USA). The following antibodies were used to stain the cell suspensions: FITC-conjugated anti-Ly-6G (clone 1A8; BD Biosciences, Franklin Lakes, NJ, USA), PE-conjugated anti-Siglec-F (clone E50-2440; BD Biosciences, Franklin Lakes, NJ, USA), and APC-conjugated anti-F4/80 (clone BM8; BioLegend, San Diego, CA, USA). BD FACSCanto II (BD Biosciences, Franklin Lakes, NJ, USA) was used to analyze the cell profiles of the BAL fluids.

### Histopathological analysis and granuloma scoring

In the PDE-challenged mice, the left lungs were excised on days 0, 2, 4, 7, and 10 after the euthanization by ketamine-xylazine overdose. In both the control mice and the PDE-challenged mice that were administered anti-IL-17A antibody, the left lung was excised on day 10 after the euthanization. On the day of lung excision, intratracheal spraying was not conducted. Each lung was inflated with 10% neutral buffered formalin and subsequently placed in formalin for 24 hours for paraffin embedding. Each lung was cut into 5-μm sections and stained with hematoxylin and eosin. The sections were evaluated under light microscopy. Ten fields from each lung section were observed at 100 × magnification, and each field was assessed individually for the numbers of the granulomata present. The mean of the numbers from each field was considered to be the granuloma score.

### Immunohistochemical analysis

We performed immunohistochemistry on the lung specimens. Paraffin-embedded 5-μm sections either were autoclaved in antigen unmasking solution (Vector Laboratories, Burlingame, CA, USA) or were digested via a protease enzyme. Frozen 8-μm sections were immersed in pre-cooled acetone for 10 min. Endogenous peroxidase was blocked with either 3% H_2_O_2_ in PBS or 0.3% H_2_O_2_ in methanol. Endogenous avidin and biotin activity were blocked with an Avidin/Biotin Blocking Kit (Vector Laboratories, Burlingame, CA, USA). The sections were incubated first with rabbit anti-mouse IL-17A polyclonal IgG (Santa Cruz Biotechnology, Dallas, TX, USA), rabbit anti-mouse CD3 polyclonal IgG (Abcam Cambridge, UK), rat anti-mouse Ly-6G monoclonal IgG (clone RB6-8C5; eBioscience, San Diego, CA, USA), or rat anti-mouse F4/80 monoclonal IgG (clone CI:A3-1; AbD Serotec, Kidlington, UK). The sections were then incubated with biotinylated secondary antibodies (goat anti-rabbit IgG or goat anti-rat IgG, Vector Laboratories, Burlingame, CA, USA). Following incubation with an ABC kit (Vector Laboratories, Burlingame, CA, USA), DAB (Nichirei, Tokyo, Japan) was added. The tissue sections were counterstained with Mayer’s hematoxylin and mounted with cover slips. Either nonimmune rabbit IgG or rat IgG was applied to the lung sections as a negative control.

### The measurement of the collagen content of the right lung

The collagen content of the right lung was determined using a Sircol Collagen Assay kit (Biocolor, Belfast, Northern Ireland, UK), according to the manufacturer’s instructions.

### An analysis of mRNA expression based on real-time quantitative polymerase chain reaction

The expression levels of the IL-17A, IL-17F, IL-6, transforming growth factor(TGF)-β1, chemokine(C-X-C motif) ligand 1 (CXCL1; KC), CXCL2 (MIP-2), CXCL5, tumor necrosis factor (TNF), interferon(IFN)-γ, IL-4, IL-5, and IL-13 genes were determined via quantitative real-time reverse transcription-polymerase chain reaction (PCR). For real-time PCR, total RNA was extracted from a portion of the right lung with TRIzol (Invitrogen, Carlsbad, CA, USA). Genomic DNA was removed using a Qiagen RNeasy kit (Qiagen, Mississauga, ON, Canada). The RNA was reverse-transcribed using a SuperScript III First-Strand Synthesis Supermix kit (Invitrogen, Carlsbad, CA, USA); oligo-dT was used as a primer. Primers were designed as Table 1. PCR was performed using a Mini Opticon (Bio-Rad, Hercules, CA, USA), operated by MJ Opticon Monitor version 3.1 analysis software (Bio-Rad, Hercules, CA, USA), using ready-made fluorogenic probes (SYBR green; Bio-Rad, Hercules, CA, USA). Gene expression was quantified relative to the expression level of ribosomal protein S15 (RPS15), using the ∆∆CT method, according to the manufacturer’s protocol.

**Table 1:** Primer sequences.

Cytokines	Forward primer	Reverse primer
IL-17A	TCATCTGTGTCTCTGATGCTGTT	TTTCCCTCCGCATTGACACA
IL-17F	AGCCATTGGAGAAACCAGCA	GGGGTCTCGAGTGATGTTGTA
IL-6	TCTCTGCAAGAGACTTCCATCC	TAACGCACTAGGTTTGCCGA
TGF-β1	AGCCCGAAGCGGACTACTAT	TCCACATGTTGCTCCACACT
CXCL1	CGAAGTCATAGCCACAC	GTGCCATCAGAGCAGTCT
CXCL2	CCCAGACAGAAGTCATAGCCA	CGAGGCACATCAGGTACGAT
CXCL5	GCATTTCTGTTGCTGTTCACGCTG	CCTCCTTCTGGTTTTTCAGTTTAGC
TNF	GCGGAGTCCGGGCAGGTCTA	GGGGGCTGGCTCTGTGAGGA
IFN-γ	TCTGGGCTTCTCCTCCTGCGG	GGCGCTGGACCTGTGGGTTG
IL-4	GGCGCTGGACCTGTGGGTTG	CCGTGCATGGCGTCCCTTCTC
IL-5	TGAGGCTTCCTGTCCCTACT	CGCCACACTTCTCTTTTTGGC
IL-13	TGCCTATGCCCTGGGGGCTC	TGGTTGCTGCCGTGGCAGAC

TGF-β1, transforming growth factor-β1; CXCL1, chemokine(C-X-C motif) ligand 1; CXCL2, chemokine(C-X-C motif) ligand 2; CXCL5, chemokine(C-X-C motif) ligand 5; TNF, tumor necrosis factor; IFN-γ, interferon-γ.

### Cytokine level measurements via an enzyme-linked immunosorbent assay

IL-17A was measured in the serum and the BAL fluids obtained from the PDE-challenged mice on days 0, 2, 4, 7 and 10 via an enzyme-linked immunosorbent assay (ELISA) (R&D Systems, Minneapolis, MN, USA).

### Statistical analysis

The results were expressed as means ± standard errors of the mean (SEs). A Mann-Whitney *U* analysis was used to determine whether there were significant differences between each group or among the PDE-challenged mice at each time point. Statistical significance was set at *P* < 0.05 bimarginally. The above-mentioned statistical analysis was performed using GraphPad PRISM (GraphPad software, San Diego, CA, USA).

## Results

### PDE enhances the lung inflammation through IL-17A

We used C57BL/6 mice to develop an acute BRHP model, because this strain is appropriate for it. Because the same dose of PDE used in C57BL/6 mice induce much weaker form of HP in 10days in Th-2 prone mice such as BALB/c mice and A/J mice (data not shown). Moreover, because the none of mice were died till 10days of post-PDE challenge, and so we considered that there was not aggressive fetal inflammation in this acute BRHP model.

**Fig 2:**
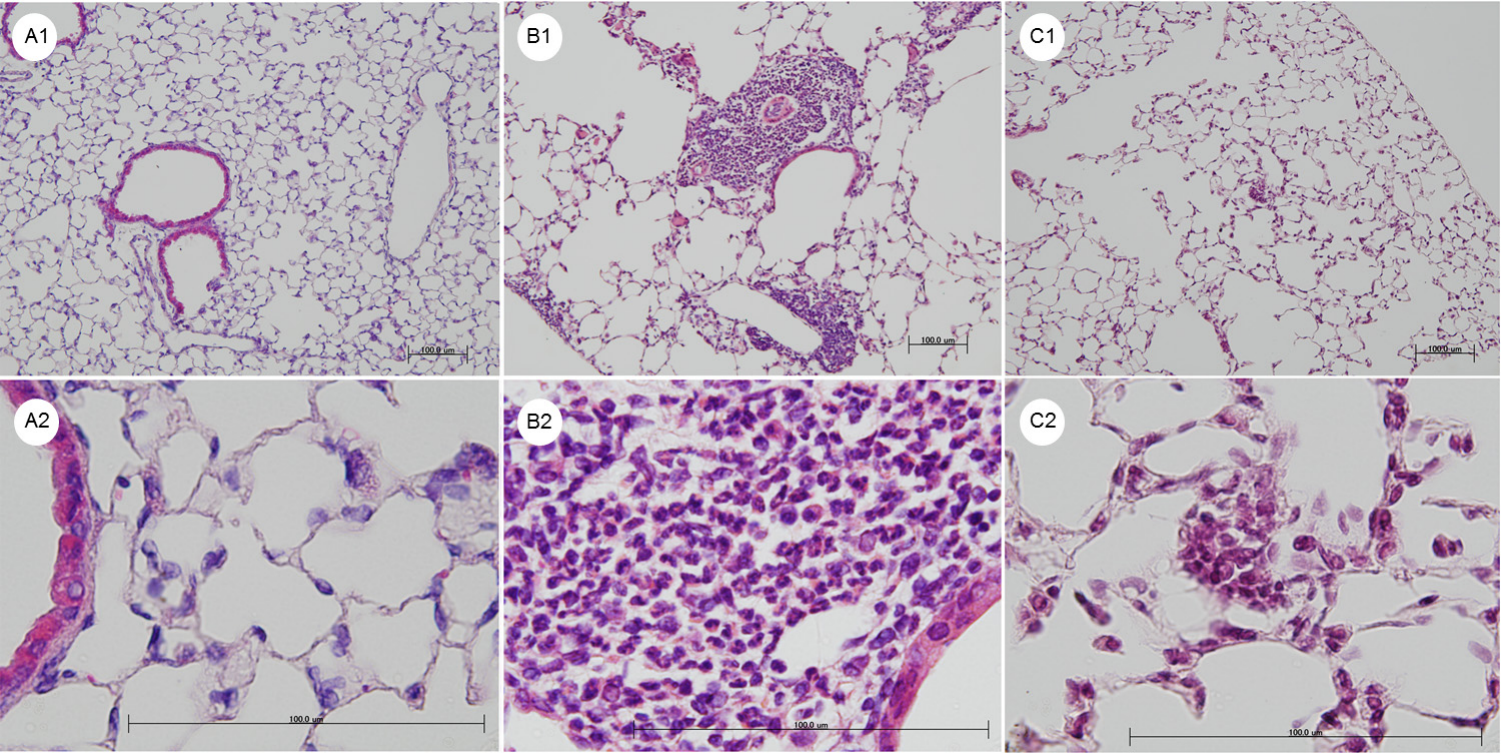
Representative hematoxylin and eosin staining of the lung sections. A1 and A2: The lungs from the control mice treated with saline for 10 days. B1 and B2: The lungs from the hypersensitivity pneumonitis (HP) model mice treated with pigeon dropping extracts (PDE) for 10 days. C1 and C2: The lungs from the PDE-challenged mice following anti-interleukin (IL)-17A antibody administration for 10 days. Intratracheal PDE administration caused inflammation and granuloma formation. Dense collections of mononuclear cells, granulomata and granulocytes are present in the photos of the PDE-challenged mice (B1 and B2). The neutralization of IL-17A decreased these inflammatory responses (C1 and C2). Shown are the 100 × (A1, B1, and C1) and 600 × (A2, B2, and C2) magnification micrographs of the original sections. Scale bars, 100 μm.

Lung histopathology revealed that PDE exposure was associated with the marked infiltration of inflammatory cells in both the peribronchiolar and perivascular areas. In the PDE-challenged mice, granulomata composed of lymphocytes, macrophages and granulocytes were noted in the peribronchiolar and perivascular areas, and many segmented granulocytes were observed in the perivascular areas, in particular (Fig 2).

**Fig 3:**
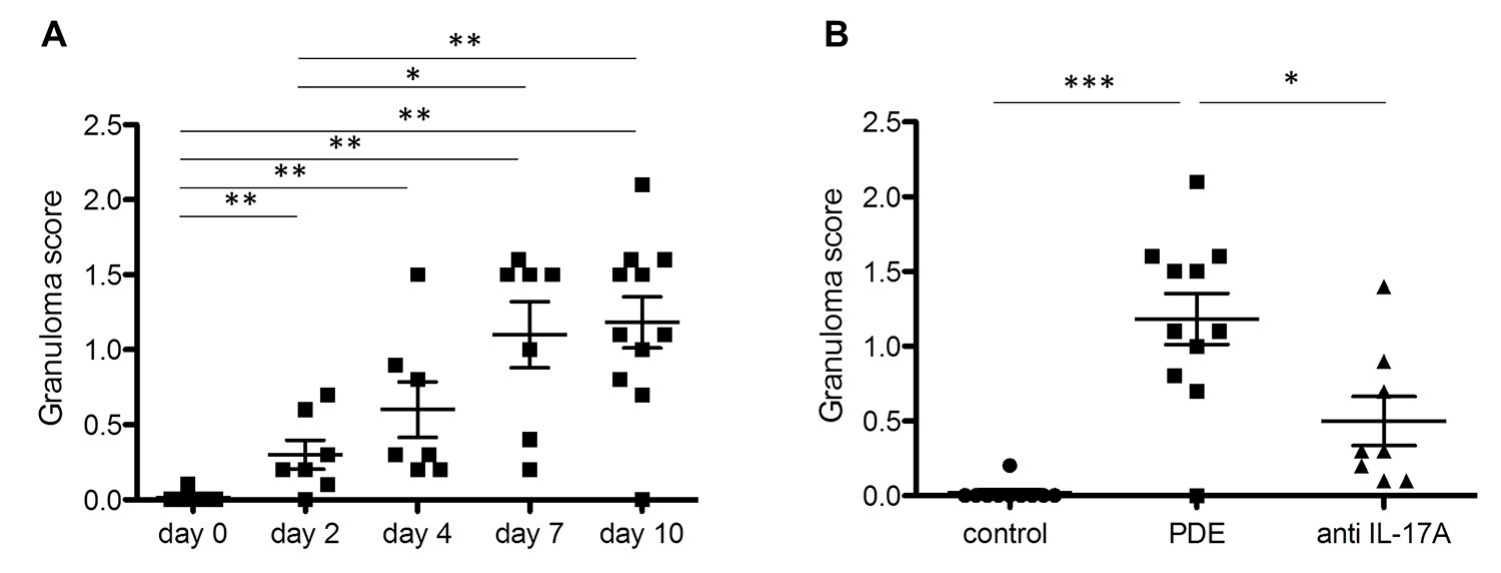
Granuloma scores. The granuloma scores were obtained by counting the numbers of granulomata individually at 100 × magnification. Each data point represented average number of granulomata from 10 microscopic fields from H&E stained lung section from one mouse. A: The granuloma scores in the pigeon dropping extract(PDE)-challenged mice. The scores on days 7 and 10 were significantly increased compared with days 0 and 2. B: The granuloma scores on day 10 in the saline-treated mice and the PDE-challenged mice, with and without interleukin(IL)-17A antibody-administration. The score in the PDE-challenged mice was significantly increased compared with that of the saline-treated mice (*P* < 0.001), and the score in the PDE-challenged mice subjected to anti-IL-17A antibody administration was significantly decreased compared with the PDE-challenged mice (*P* = 0.021). The data are presented as the means ± standard errors of the mean (SEs) for each group (day 0–10: n = 7–11, control group: n = 10, PDE group: n = 11, anti-IL-17A group: n = 8). *: *P* < 0.05, **: *P* < 0.01, ***: *P* < 0.001.

The granuloma scores of the PDE-challenged mice on days 7 and 10 were significantly increased compared with days 0 and 2 (day 0: 0.01 ± 0.01, day 2: 0.30 ± 0.10, day 4: 0.60 ± 0.19, day 7: 1.10 ± 0.22, day 10: 1.18 ± 0.17; Fig 3A). The granuloma score in the PDE-challenged mice was significantly increased on day 10 compared with the control mice (control mice: 0.02 ± 0.02, PDE-challenged mice: 1.18 ± 0.17; *P* < 0.001; Fig 3B), and the score in the PDE-challenged mice that were administered the anti-IL-17A antibody was significantly decreased compared with the PDE-challenged mice (PDE-challenged mice administered the anti-IL-17A antibody: 0.50 ± 0.16; *P =* 0.021; Fig 3B).

**Fig 4:**
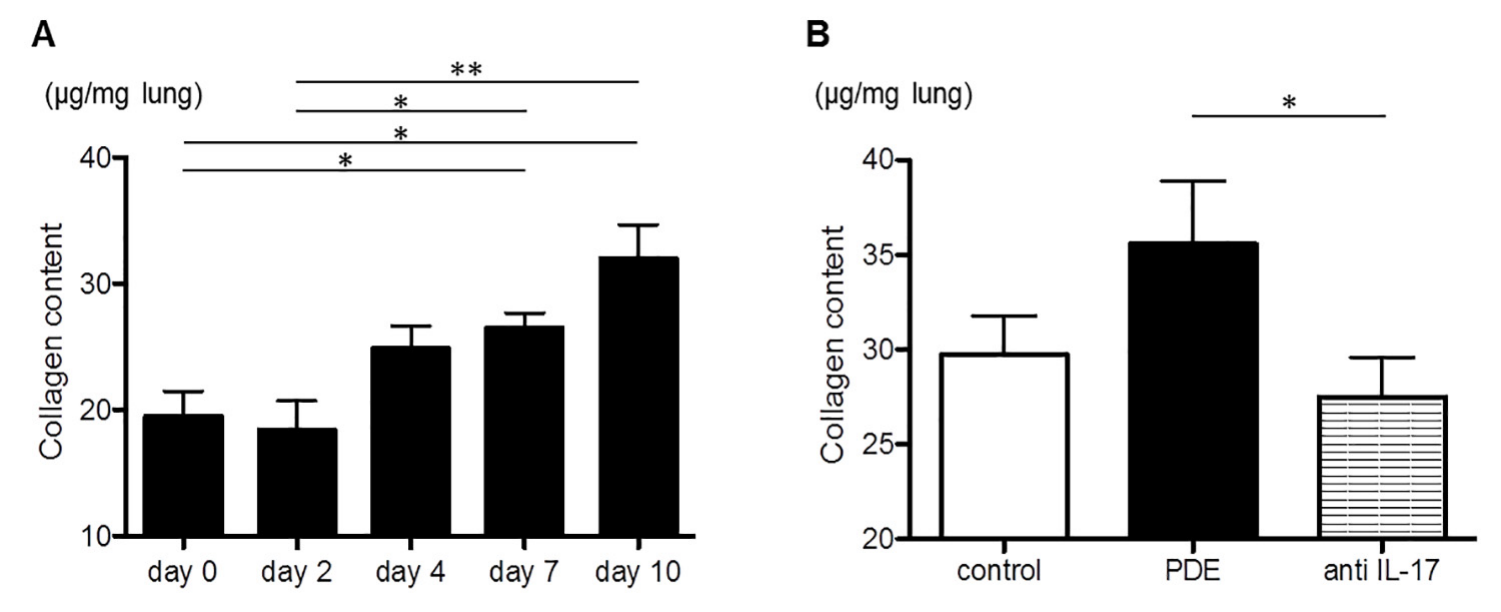
An evaluation of the inflammatory changes via measurements of the collagen content of the lungs. A: The collagen content of the pigeon dropping extract(PDE)-challenged mice. The values on days 7 and 10 were significantly increased compared with days 0 and 2. B: The collagen contents on day 10 in the saline-treated mice and the PDE-challenged mice, with and without anti-interleukin(IL)-17A antibody administration. The values among the PDE-challenged mice were not significantly different compared with those of the saline-treated mice, whereas the values of the PDE-challenged mice subjected to anti-IL-17A antibody administration were significantly decreased compared with the PDE-challenged mice (*P* = 0.029). The data are presented as means ± standard errors of the mean (SEs) for each group (day 0–10: n = 7–11, control group: n = 10, PDE group: n = 11, anti-IL-17A group: n = 8). *: *P* < 0.05, **: *P* < 0.01.

The collagen content of the right lung in the PDE-challenged mice on days 7 and 10 was significantly increased compared with days 0 and 2 (day 0: 19.5 ± 2.0, day 2: 18.4 ± 2.3, day 4: 24.9 ± 1.8, day 7: 26.5 ± 1.2, day 10: 32.0 ± 2.7 μg/mg lung; *P* = 0.005; Fig 4A). The collagen content of the PDE-challenged mice was not significantly different (control mice: 29.7 ± 2.0, PDE-challenged mice: 35.6 ± 3.3 μg/mg lung; *P* = 0.219; Fig 4B), but the collagen content of the PDE-challenged mice that were administered the anti-IL-17A antibody was significantly decreased compared with the PDE-challenged mice (PDE-challenged mice administered the anti-IL-17A antibody: 27.5 ± 2.1 μg/mg lung; *P* = 0.029; Fig 4B).

### The expression level of CXCL5 in the lungs was decreased by anti-IL-17A antibody

**Fig 5:**
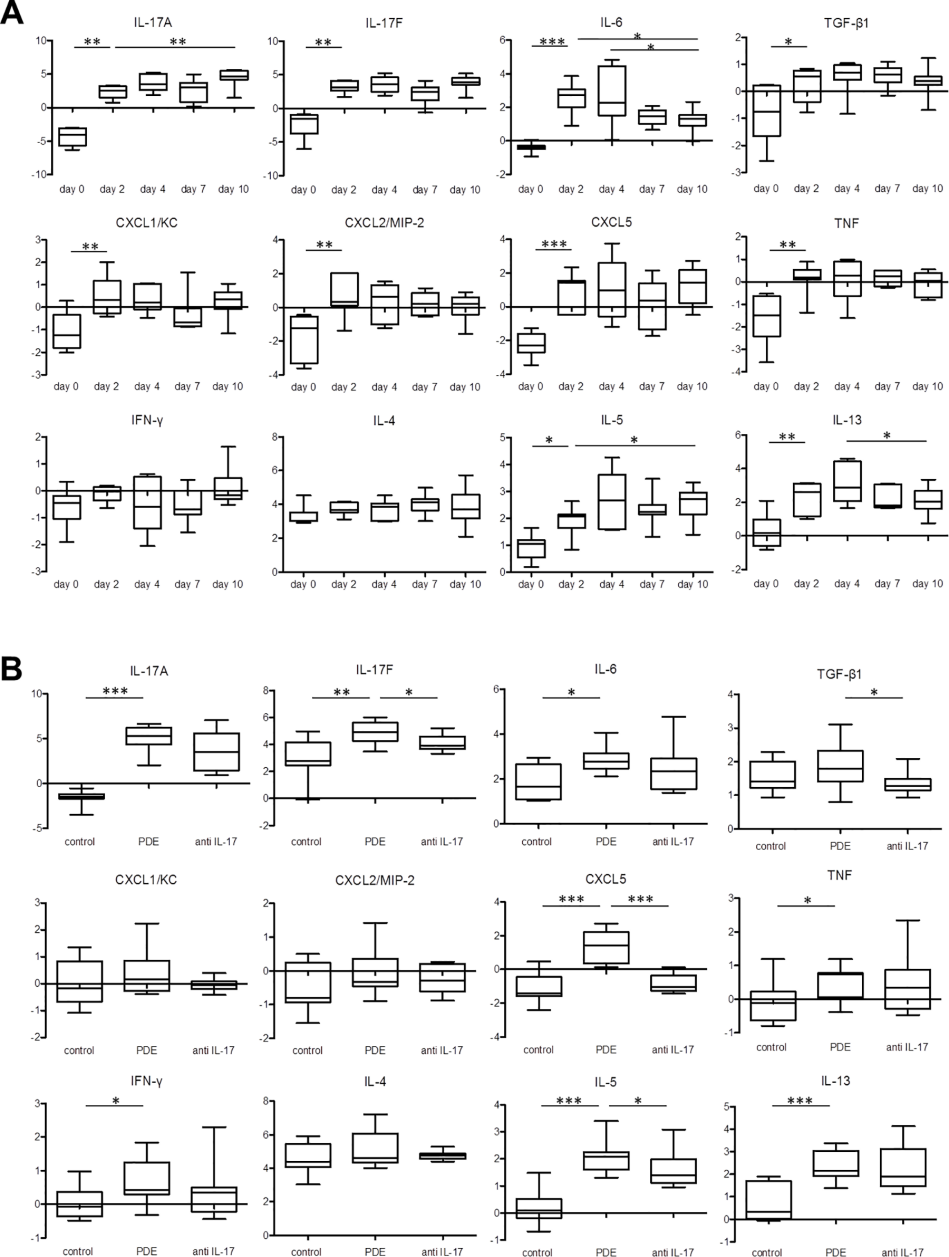
The presence of mRNA in each lung sample as determined via real-time PCR. The levels of mRNA for interleukin(IL)-17A, IL-17F, IL-6, transforming growth factor(TGF)-β1, chemokine(C-X-C motif) ligand 1 (CXCL1; KC), CXCL2 (MIP-2), CXCL5, tumor necrosis factor (TNF), interferon(IFN)-γ, IL-4, IL-5, and IL-13 or ribosomal protein S15 (RPS15) (internal control) were each measured. Gene expression was quantified relative to the level of RPS15, using the ∆∆CT method. The Y-axis was -∆∆CT which meant the difference of the ∆CT between target samples and control samples those were lungs in an untreated C57BL/6 female mouse. The ∆CT meant the difference of the CT value between target genes and endogenous control gene which was RPS15 in the current study. The CT value was the number of cycles that it took each reaction to reach an arbitrary amount of fluorescence. A: The data from the pigeon dropping extract(PDE)-challenged mice. The expression levels of IL-17A and IL-5 mRNA on day 10 were significantly increased compared with those obtained on day 2, when only a few granulomata were present. Only the expression level of IL-6 mRNA was significantly decreased on day 10 compared with day 2. B: The data on day 10 from the saline-treated mice and the PDE-challenged mice, with or without anti-IL-17A antibody administration. The expression levels of IL-17A, IL-17F, IL-6, CXCL2, CXCL5, TNF, IFN-γ, IL-5, and IL-13 mRNA were significantly increased in the PDE-challenged mice compared with the control mice. The expression levels of IL-17F, TGF-β1, CXCL5 and IL-5 mRNA were significantly decreased in the PDE-challenged mice subjected to anti-IL-17A antibody administration compared with the PDE-challenged mice. The data are presented as means ± standard errors of the mean (SEs) for each group (day 0–10: n = 7–11, control group: n = 10, PDE group: n = 11, anti-IL-17A group: n = 8). *: *P* < 0.05, **: *P* < 0.01, ***: *P* < 0.001.

The expression levels of every mRNA except IFN-γ and IL-4 were significantly increased on day 2 compared with day 0 (Fig 5A). The expression levels of IL-17A and IL-5 mRNA were significantly increased on day 10 compared with day 2 (Fig 5A). Only the expression level of IL-6 mRNA was significantly decreased on day 10 compared with day 2 (Fig 5A). The expression levels of IL-6 and IL-13 mRNA peaked on days 2 and 4, and the levels noted on day 10 were decreased compared with day 4 (Fig 5A). The expression levels of IL-17A, IL-17F, IL-6, CXCL2, CXCL5, TNF, IFN-γ, IL-5, and IL-13 mRNA were significantly increased in the PDE-challenged mice compared with the control mice (Fig 5B). The expression levels of IL-17F, TGF-β1, CXCL5 and IL-5 mRNA were significantly decreased in the PDE-challenged mice that were administered the anti-IL-17A antibody compared with the PDE-challenged mice (Fig 5B).

**Fig 6:**
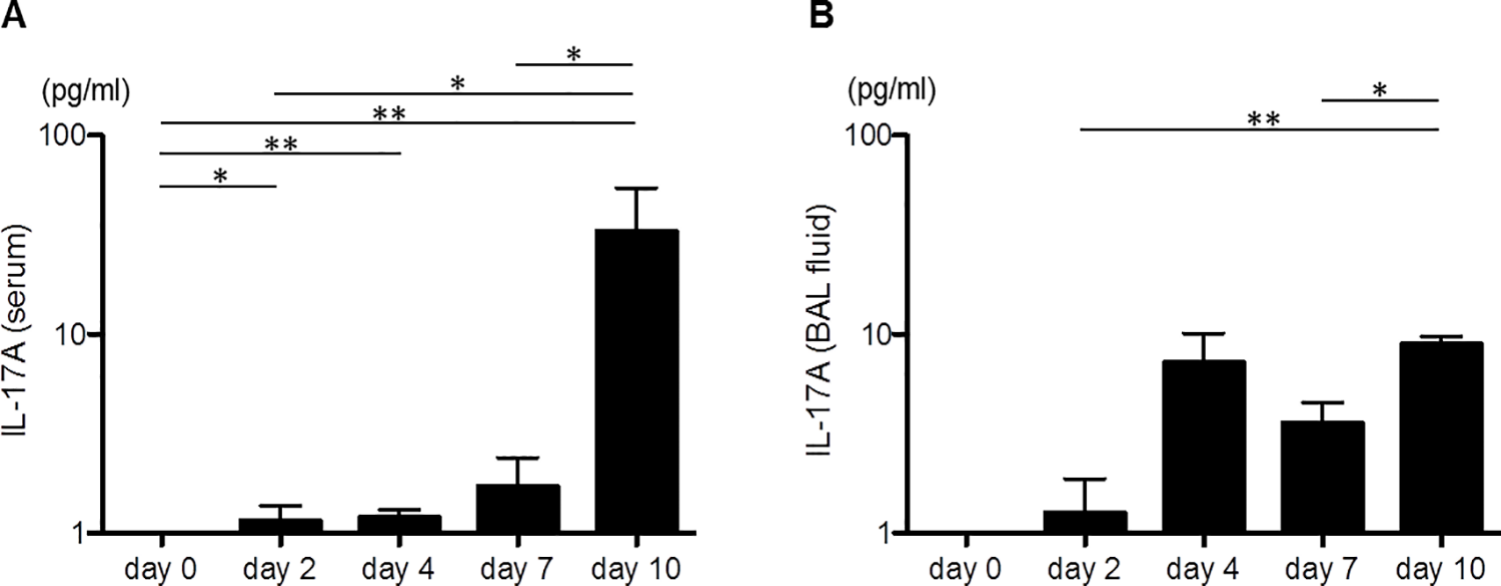
The interleukin(IL)-17 protein levels obtained from the pigeon dropping extract(PDE)-challenged mice. A: The interleukin(IL)-17A levels in the serum. The levels on day 2 were significantly increased compared with day 0 (*P* = 0.032), and the levels on day 10 were significantly increased compared with day 2 (*P* = 0.022). B: The IL-17A levels in the bronchoalveolar lavage (BAL) fluids. The levels on day 10 were significantly increased compared with day 2 (*P* = 0.006). The data are presented as means ± standard errors of the mean (SEs) for each group (day 0–10: n = 7–11, control group: n = 10, PDE group: n = 11, anti-IL-17A group: n = 8). *: *P* < 0.05, **: *P* < 0.01.

The IL-17A expression levels in the serum and the BAL fluids of the PDE-challenged mice were significantly increased on day 10 compared with day 2, when only a few granulomata were present (serum; day 2: 1.16 ± 0.22, day 10: 32.9 ± 21.6 pg/ml; *P* = 0.022, BAL fluids; day 2: 1.27 ± 1.61, day 10: 8.94 ± 0.77 pg/ml; *P* = 0.006; Fig 6).

### Siglec-F^int^ Ly-6G^high^ F4/80^neg^ cells in BAL fluids increased in an early phase of the PDE-challenged model and decreased by anti-IL-17A antibody

**Fig 7:**
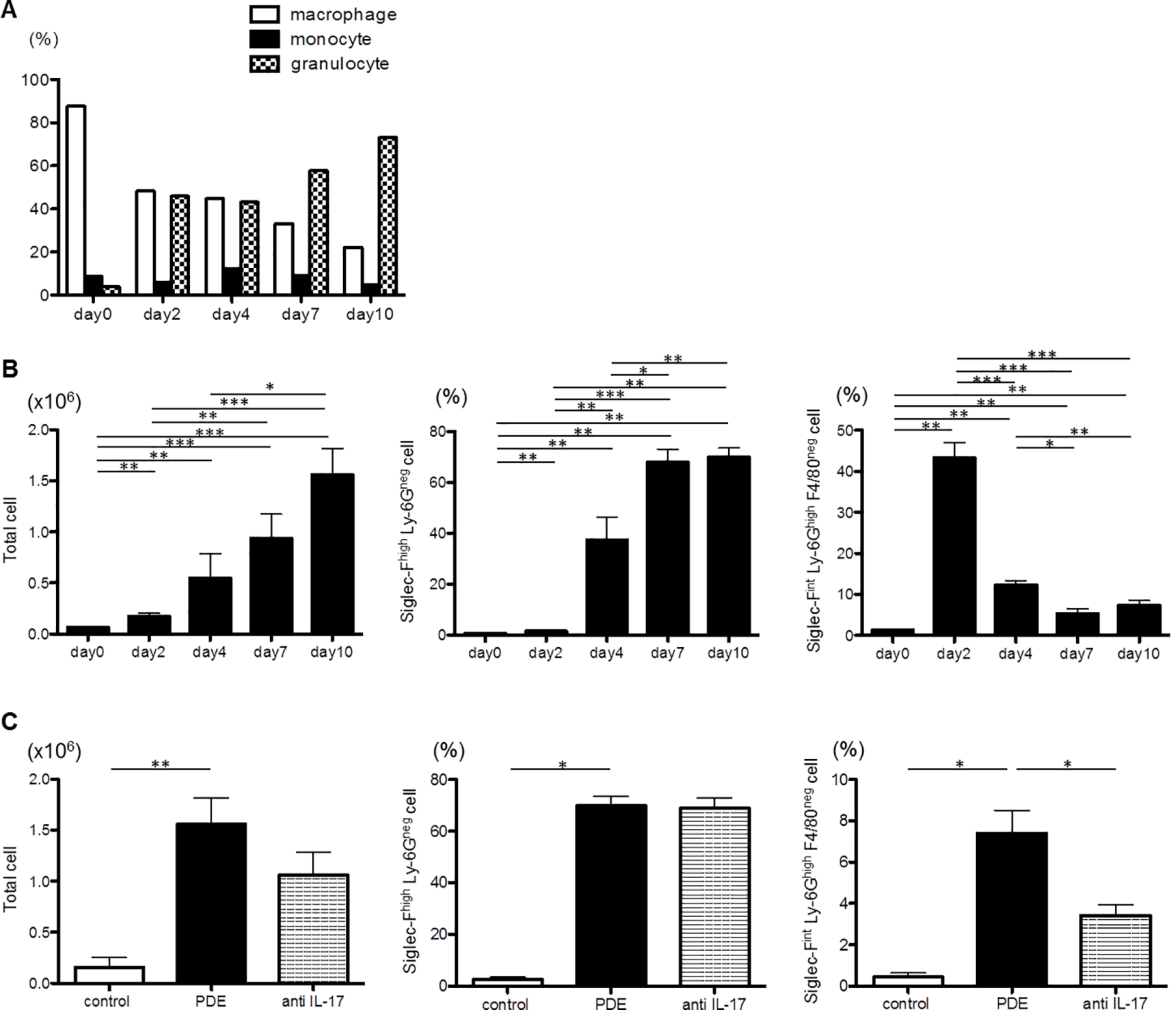
The inflammatory cell levels in the bronchoalveolar lavage fluids. A: The ratios of macrophages, monocytes and granulocytes in the bronchoalveolar lavage (BAL) fluids of the pigeon dropping extract(PDE)-challenged mice as determined via Diff-Quick staining. B: The total cell numbers and the ratios of Siglec-F^high^ Ly-6G^neg^ cells and Siglec-F^int^ Ly-6G^high^ F4/80^neg^ cells in the BAL fluids of the PDE-challenged mice as determined via flow cytometric analysis. The total cell number and the ratio of Siglec-F^high^ Ly-6G^neg^ cells on day 2 were significantly increased compared with day 0, and both the number and the ratio on day 10 were significantly increased compared with day 2. The ratio of Siglec-F^int^ Ly-6G^high^ F4/80^neg^ cells peaked on day 2, and the ratios on days 4, 7 and 10 were significantly decreased compared with day 2. C: The total cell number and the ratios of Siglec-F^high^ Ly-6G^neg^ cells and Siglec-F^int^ Ly-6G^high^ F4/80^neg^ cells on day 10 in the BAL fluids of the saline-treated mice and the PDE-challenged mice, with and without anti-interleukin(IL)-17A antibody administration as determined via flow cytometric analysis. The total cell number and the ratios of Siglec-F^high^ Ly-6G^neg^ cells and Siglec-F^int^ Ly-6G^high^ F4/80^neg^ cells of the PDE-challenged mice were significantly increased compared with the saline-treated mice. The total cell number and the ratio of Siglec-F^high^ Ly-6G^neg^ cells of the PDE-challenged mice subjected to anti-IL-17A antibody administration were not significantly different compared with the PDE-challenged mice, but only the ratio of Siglec-F^int^ Ly-6G^high^ F4/80^neg^ cells was significantly decreased. The data are presented as means ± standard errors of the mean (SEs) for each group (day 0–10: n = 7–11, control group: n = 10, PDE group: n = 11, anti-IL-17A group: n = 8). *: *P* < 0.05, **: *P* < 0.01, ***: *P* < 0.001.

The numbers of total cells, monocytes and granulocytes in the BAL fluids of the PDE-challenged mice increased each time PDE was sprayed (Fig 7A and 7B). The ratio of granulocytes also increased significantly, whereas the ratio of monocytes did not (Fig 7A). The numbers of total cells and the ratios of Siglec-F^high^ Ly-6G^neg^ cells and Siglec-F^int^ Ly-6G^high^ F4/80^neg^ cells in the BAL fluids of the PDE-challenged mice were significantly increased on day10 compared with the control mice (total cells; control mice: 0.15 ± 0.09 × 10^6^ cells, PDE-challenged mice: 1.56 ± 0.26 × 10^6^ cells; *P* = 0.005, Siglec-F^high^ Ly-6G^neg^ cells; control mice: 2.5 ± 1.0%, PDE-challenged mice: 69.8 ± 3.7%; *P* = 0.017, Siglec-F^int^ Ly-6G^high^ F4/80^neg^ cells; control mice: 3.43 ± 0.52%, PDE-challenged mice: 0.43 ± 0.20%; *P* = 0.017; Fig 7B). The numbers of total cells and the ratio of Siglec-F^high^ Ly-6G^neg^ cells in the BAL fluids of the PDE-challenged mice that were administered the anti-IL-17A antibody were not significantly different on day10 compared with the PDE-challenged mice (total cells; PDE-challenged mice administered the anti-IL-17A antibody: 1.06 ± 0.22 × 10^6^ cells; *P* = 0.557, Siglec-F^high^ Ly-6G^neg^ cells; PDE-challenged mice administered the anti-IL-17A antibody: 68.9 ± 4.0%; *P* = 0.788; Fig 7C). The ratio of Siglec-F^int^ Ly-6G^high^ F4/80^neg^ cells in the BAL fluids of the PDE-challenged mice that were administered the anti-IL-17A antibody was significantly decreased on day 10 compared with the PDE-challenged mice (PDE-challenged mice administered the anti-IL-17A antibody: 3.43 ± 0.52%; *P* = 0.042; Fig 7C). The ratio of Siglec-F^int^ Ly-6G^high^ F4/80^neg^ cells in the BAL fluids of the PDE-challenged mice was significantly decreased on day 10 compared with day 2, when only a few granulomata were present (day 10: 7.4 ± 1.1%, day 2: 40.4 ± 3.9%; *P* < 0.001; Fig 7B).

### Most of neutrophils in the granulomata were stained with the anti-IL-17A antibody

**Fig 8:**
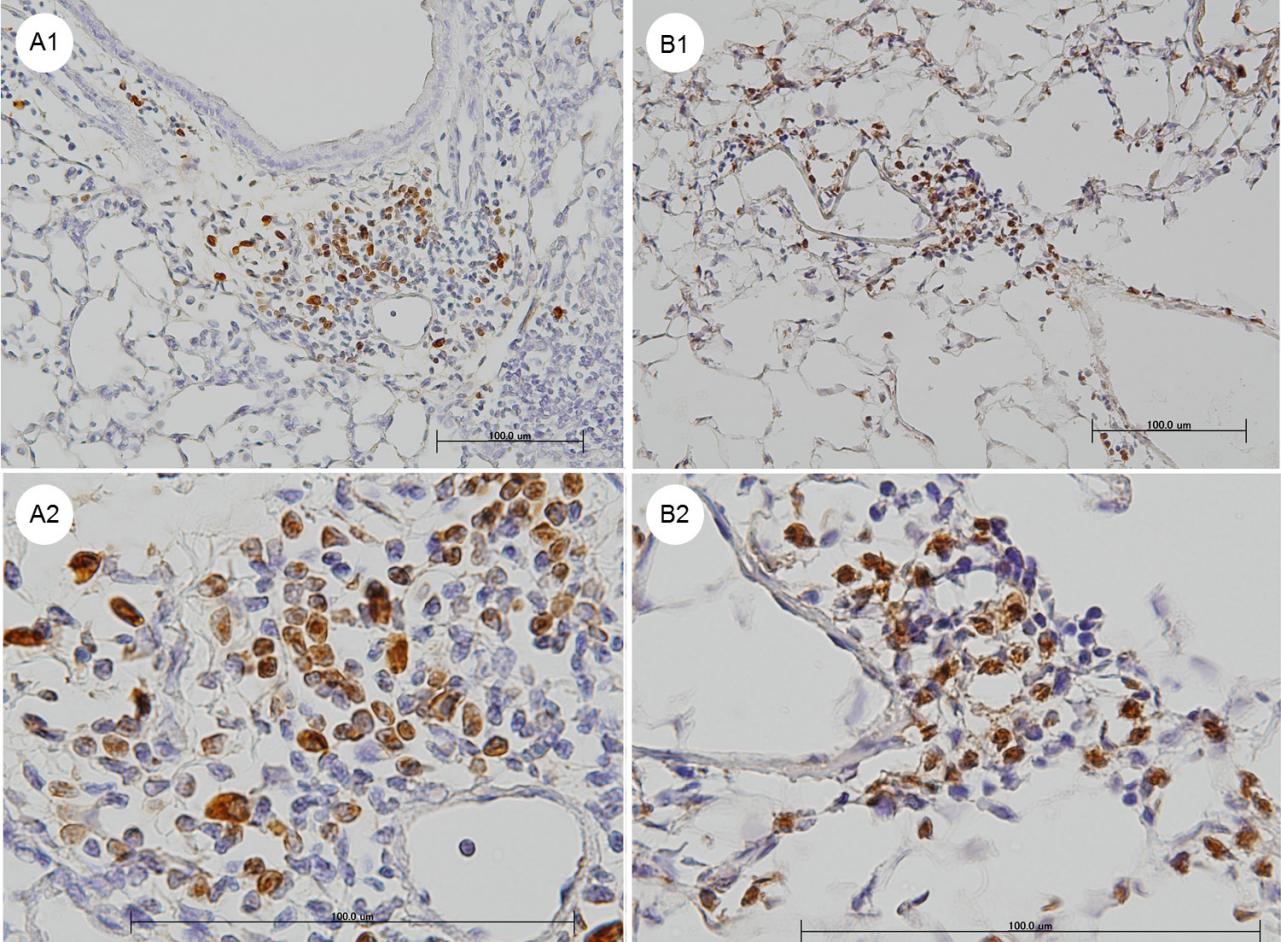
Representative pictures of immunohistochemical staining with an anti-Ly-6G antibody and an anti-interleukin(IL)-17A antibody. The lungs from the hypersensitivity pneumonitis (HP) model mice that were treated with pigeon dropping extract (PDE) for 10 days. A1 and A2: Staining with the anti-Ly-6G antibody. Most of the segmented granulocytes were stained with the anti-Ly-6G antibody. B1 and B2: Staining with the anti-IL-17A antibody. Most of the cells stained with the anti-Ly-6G antibody were also stained with anti-IL-17A antibody. Shown are the 200 × (A1 and B1) and 600 × (A2 and B2) magnification micrographs of the original sections. Scale bars, 100 μm.

An immunohistochemical analysis of the lungs of the PDE-challenged mice revealed that most of the monocytes in the granulomata were stained with anti-CD3 antibody, whereas the segmented granulocytes and macrophages were not stained with this antibody. Most of the segmented granulocytes were stained with the anti-Ly-6G antibody (Fig 8A), and most of these cells were also stained with the anti-IL-17A antibody (Fig 8B).

## Discussion

The repeated exposure to the PDE antigen in our model of HP resulted in the marked infiltration of inflammatory cells and the formation of granulomata composed of lymphocytes, macrophages and segmented granulocytes in the peribronchiolar and perivascular areas (Fig 2B). The segmented granulocytes were assumed to be neutrophils, because most of the cells were stained with the anti-Ly-6G antibody (Fig 8A) and Ly-6G is reportedly highly expressed in neutrophils [23]. The ratio of Siglec-F^int^ Ly-6G^high^ F4/80^neg^ cells in the BAL fluids peaked on day 2 but was decreased on day 10 in the PDE-challenged mice. The Siglec-F^int^ Ly-6G^high^ F4/80^neg^ cells were assumed to be neutrophils, because it has been reported that Ly-6G is highly expressed in neutrophils [23], and Siglec-F is highly expressed in both eosinophils and alveolar macrophage and is weakly expressed on neutrophils in the lungs [24–26]. In humans, neutrophilic infiltrates have also been described [27]. Neutrophils were the predominate cell type noted in the BAL fluids 24 hours following the challenge with the inciting antigen [28]. In our model of acute HP, it was observed that the neutrophils played an important role in the immunological response involving granulomata formation, particularly in an early phase of the process.

The collagen content and the expression levels of IL-17A mRNA in the lungs and IL-17A protein in the serum and the BAL fluids were significantly increased. On day 2 in our acute HP model, only a few granulomata were present, and the collagen content in the lungs was not increased. However, the expression levels of many cytokine mRNA molecules in the lungs were already increased on day 2. The numbers of granulocytes in the BAL fluid were increased on day 2, and the ratio of neutrophils in the BAL fluid peaked on day 2. The expression level of IL-6 mRNA in the lungs peaked on days 2 and 4, but was decreased at intervals thereafter. Therefore we surmised that IL-6 and IL-17A were responsible for the response, including the neutrophilic infiltrates noted during the early phase of the response.

In the PDE-challenged mice that were administered the anti-IL-17A antibody on day 10, the granuloma score and the collagen content in the lungs were each significantly decreased compared with the PDE-challenged mice. The neutralization of IL-17A by the antibody significantly decreased the expression level of CXCL5 (*P* < 0.001), and also significantly decreased the ratio of Siglec-F^int^ Ly-6G^high^ F4/80^neg^ cells, which were assumed to be neutrophils, in the BAL fluids (*P* = 0.042). Most of the segmented granulocytes were stained with the anti-Ly-6G antibody (Fig 8A), and most of these cells were also stained with the anti-IL-17A antibody (Fig 8B). Therefore, the neutrophil accumulation in the granuloma was assumed to be the source of IL-17A, and CXCL5 was assumed to be a downstream chemokine in IL-17A signaling.

**Fig 9:**
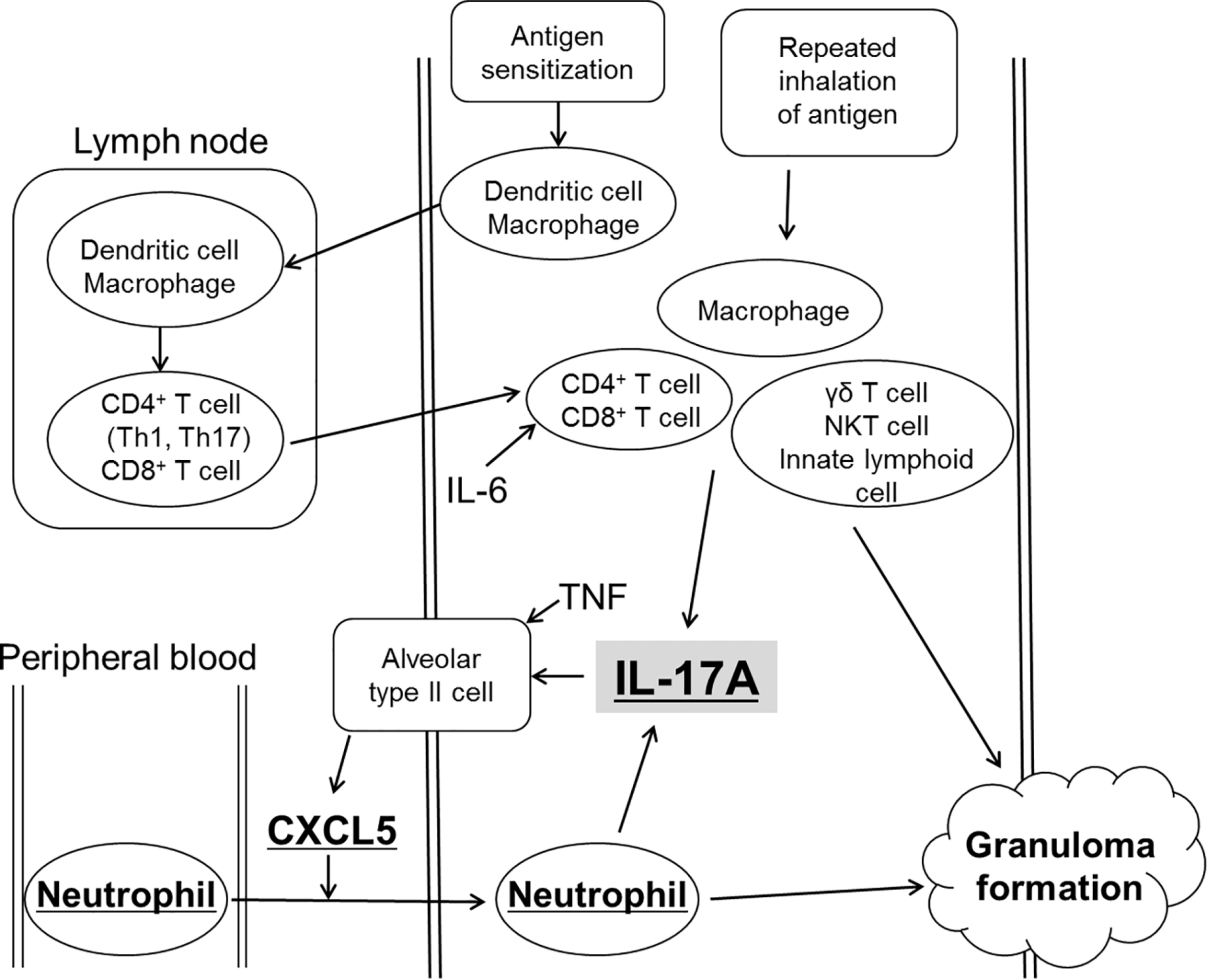
The roles of interleukin(IL)-17A in granuloma formation in our murine model of hypersensitivity pneumonitis. The repeated inhalation of the antigens by sensitized-mice stimulated the secretion of the IL-17A cytokine by CD4^+^ T cells, CD8^+^ T cells, macrophages, γδ T cells, NKT cells, and innate lymphoid cells. IL-6 stimulated secretion by Th17 cells. IL-17A and TNF increased the secretion of CXCL5, which accelerated the recruitment of neutrophils by alveolar type II cells. The neutrophils also secreted IL-17A. The neutrophils accumulated together with lymphocytes, macrophages and giant cells, and formed granulomatous lesions. IL-17A, CXCL5 and neutrophils each played important roles in granuloma formation.

CD4^+^ T cells [29,30], CD8^+^ T cells [31,32], γδ T cells [33], NKT cells [34], innate lymphoid cells [35], macrophages [36], and neutrophils [37,38] were each noted as cellular sources of IL-17A. The intratracheal coadministration of IL-17A and TNF in mice induced the production of CXCL1, CXCL2 and CXCL5, and the source of CXCL5 was cells residing in the lungs [39]. In hypersensitivity pneumonitis, the effects of IL-17A were dependent on signaling in the structural cells, but not in the bone marrow-derived cells [40]. In an LPS-challenged murine model, alveolar epithelial type II cells (AT II cells) were reportedly the primary source of CXCL5 in the lungs [41]. Human AT II cells secrete CXCL5 in order to recruit neutrophils to assist in host defense in the setting of an initial injury [39]. In a murine arthritis model, the neutralization of CXCL5 ameliorated joint inflammation, bone destruction and vascularization [42]. In our model of acute HP, the administration of the anti-IL-17A antibody decreased the formation of granulomata and the ratio of neutrophils in the BAL fluids, and decreased the expression level of CXCL5 in the lungs. These results indicate that CXCL5 is an important downstream mediator of IL-17A, and that both IL-17A and CXCL5 are responsible for the recruitment of neutrophils (Fig 9). Based on the evidence above, IL-17A is secreted from CD4^+^ T cells, CD8^+^ T cells, γδ T cells, NKT cells, innate lymphoid cells, macrophages and neutrophils by the repeated inhalation of antigen, and then assumed IL-17A stimulated AT II cells which secreted CXCL5, and the increase in CXCL5 expression stimulated the recruitment of neutrophils to the lungs. Because the results in the current study suggest that neutrophils could secrete IL-17A, we consider that the IL-17A-CXCL5 pathway plays a key role in the amplification of inflammation in an early phase of acute HP.

One of the limitation of the present study is the method of depleting the IL-17A cytokine. We cannot fully rule out the possibility that the anti-IL-17A antibody influenced the surrounding environment. Another of the limitation of the study is lack of results whether inflammation was attenuated in neutrophil-depleted mice. We tried to deplete neutrophils by anti-Gr-1 antibody to prove that neutrophils play an important role and secretethe IL-17A cytokine in acute HP. Hasan SA et al depleted neutrophils in SR-induced HP model mice by injected 100 μg of anti-Gr-1 antibody twice a week, which was the same product (eBioscience, San Diego, Calif; clone RB6-8C5) as we used intraperitoneally [40]. However we failed to deplete neutrophils in the PDE-challenged acute HP model mice by injected 200 μg of the antibody thrice a week, as we considered that neutrophils were stimulated more aggressively in the present model than SR-induced model reported in the past. Certainly, more studies utilizing neutrophil-transgenic mice and IL-17A-transgenic mice are necessary.

In summary, the results in the current study indicated that both the IL-17A–CXCL5 pathway and neutrophils play important roles in granuloma formation in the setting of acute HP and the increase in IL-17A expression stimulates the recruitment of neutrophils to the lungs via the IL-17A–CXCL5 pathway. We assumed that these neutrophils secrete the IL-17A cytokine. These findings have provided us with new insights into the mechanisms underlying granuloma formation in the setting of acute HP, and have identified new potential therapeutic targets such as IL-17A and CXCL5.
